# Absence of SARS-CoV-2 in the air and on the surfaces within the school environment

**DOI:** 10.1099/jmm.0.001424

**Published:** 2021-09-20

**Authors:** Amit Thakar, Shawn Dutkiewicz, Timothy Hoffman, Paul Joyce, Vishal Shah

**Affiliations:** ^1^​ University of Wyoming, Laramie, WY, 82071, USA; ^2^​ Radnor Township School District, Wayne, PA, 19087, USA; ^3^​ Unionville Chadds-Ford School District, Kennett Square, PA, 19348, USA; ^4^​ West Chester Area School District, Exton, PA, 19341, USA; ^5^​ West Chester University, West Chester, PA, 19383, USA

**Keywords:** air, coronavirus, fomite, surface, schools, SARS-CoV-2

## Abstract

To the best of our knowledge to date there are no scientific studies specifically investigating whether the SARS-CoV-2 virus is present in the air or on the various surfaces in the school environment. The aim of this study was to determine if SARS-CoV-2 is present on various high touch surfaces and in the air across the elementary, middle and high schools in the Chester County of Pennsylvania, USA. One hundred and fifty surface swab samples and 45 air samples were analysed for the presence of the virus. All the samples tested were negative for the presence of SARS-CoV-2. The results indicate that the spread of the virus through contact and through air in the school buildings across the USA is highly unlikely.

## Introduction

Current guidance from the Centres for Disease Control and Prevention (CDC), Atlanta, Georgia, USA states that schools can be reopened with mitigating measures in place to prevent the spread of SARS-CoV-2. The updated guidance includes epidemiological data on the transmission rates within schools providing in-person instructions in the United States and other countries [1, 2]. With widespread vaccination of Kindergarten through 12th grade (K-12) teachers, school staff, and adolescent students 12–17 years old, the school environment can be presumed to be much safer than many of the community settings with lower fraction of the population being vaccinated [3]. Nevertheless, there remains reluctance and fear within the society concerning the role of in-person K-12 education in spreading the virus [4]. Of the many reasons contributing to the reluctance of in-person tuition amongst students and parents, one of them is the lack of scientific studies investigating the presence of SARS-CoV-2 virus in the school environment while students are receiving in-person tuition. In our study we looked for the presence of the virus in the air and on surfaces in nine schools across the Chester County, PA. The aim of the study was to determine whether or not SARS-CoV-2 could be detected in the air or on surfaces in the school buildings which had the recommended mitigation measures in place.

## Methodology

Air and surface swab samples were collected from an elementary, middle and high school from three school districts in Chester County, PA, between the second week of April and first week of May 2021. During this duration, the county had a qPCR positivity rate of >5.0 % and was in the peak of the Spring wave of numbers of new COVID-19 cases reported per day. The daily new cases within the county for the entire population ranged from 38 to 73 during sampling duration. Average number of students and faculty combined in elementary, middle and high schools were 403, 775, and 1063, respectively. Fifteen swab samples were collected from each elementary and middle school and 20 swab samples were collected from each of the high schools. The swab samples were collected immediately at the end of the school day once the students were dismissed. The locations were primarily high touch points such as hand railings, desks, and door handles. Air samples were collected from locations including classrooms, cafeterias, and hallways using M-TRAP air filter cassettes and WhisperCare air pumps from AssuredBio Labs, Tennessee. The cassettes and pumps combination from the manufacturer are used commercially for the detection of SARS-CoV-2 in the air samples. Air samples were collected by operating the pump for the entire duration of the school session (minimum of 6 h). The RNA was extracted from the surface swab or air filter by submerging them in a 200 µl One-step DNA/RNA Extraction buffer (CHAI, CA, USA) and rotating for at least ten times to release the sample cells into the extraction buffer (as recommended by the manufacturer). The RNA extracts were subjected to RT-PCR using a Coronavirus Environmental Test Kit (CHAI, CA, USA) to detect the presence of SARS-CoV-2. To validate the methodology, doorknobs, door handles, and desk surfaces were spiked with 5 µl SARS-CoV-2 N positive control provided in the kit. Swabbing, extraction, and detection were performed using methods described above to confirm if SARS-CoV-2 was detected. In all the validation results, positive results were only obtained when the surfaces were spiked with positive control. Detection limit of the kit used is 290 viral copies per swab.

## Results and discussion

Of the 150 surfaces analysed from nine schools, no samples showed the presence of the virus on the surfaces analysed. The surfaces analysed varied widely and ranged from areas sanitized frequently such as desks, cafeteria and teaching tables, musical instruments, and classroom door handles to areas not frequently sanitized such as vending machines, towel dispensers, handrails, lockers, photocopiers, appliances, school main entrance doors, and sports and gymnasium equipment. While increased surface cleaning has been undertaken by all the school buildings studied, the lack of SARS-CoV-2 on the surfaces that are not frequently sanitized supports the recent CDC guidelines suggesting a low risk of the virus being spread through surface transmission [5]. The confidence is further increased from the fact that the swab samples were collected immediately after school dismissal, before the end-of-day disinfection cleanup was initiated.

The SARS-CoV-2 virus was not detected in all 45 of 45 (100 %) air samples. Prior to opening the schools for in-person tuition, the ventilation systems in all the buildings were modified to minimize the spread of the virus and based on CDC recommendations. The modifications were based on the building layout and the age of the ventilation systems. Examples of the changes made in the ventilation system includes operating the systems at highest efficiency (e.g. operating it for 24 h/7 days a week, increasing the number of air changes per hour), changing the air filters at a higher frequency, setting the air dampers to 100 % open when weather and indoor quality factors allow for this to be possible. Some buildings also closed doors and windows to allow systems to operate under negative pressure, stopped operation of ceiling fans to ensure designed air circulation patterns are maintained, and added portable High Efficiency Particulate Air (HEPA) purifiers to classrooms and office spaces to increase air exchanges. Also, all students, teachers and staff were required to wear face masks when inside the buildings. Acknowledging the possibility of asymptomatic cases that may remain undetected, the absence of SARS-CoV-2 in the air provides evidence that modifications to air ventilation system and face masking are effective in preventing the spread of the virus through air. The results are supported by the scenario-based analysis reported by Xu *et al*. showing decreased risk of airborne SARS-CoV-2 when modifications to ventilation systems are made [6].

As of August 2021, the United States is seeing an increase in the number of cases of COVID-19 within the community. The rise is fueled by the spread of the delta variant of SARS-CoV-2. The variant has been shown to infect a younger population (<24 years old), particularly the population within this group that is not vaccinated [7]. The current study was conducted when the delta variant was not prevalent in the community. While further studies are warranted to confirm if the results hold true for the delta variant, we believe that schools having modified air ventilation systems and having face mask requirements should be considered as safe environments and present minimal risk to students, teachers, and staff. To reduce any chances of exposure to SARS-CoV-2 possibly present on surfaces, schools should continue to encourage using hand sanitizer or washing hands periodically.
